# Literary and Journalistic Notes

**Published:** 1904-12

**Authors:** 


### LITERARY AND JOURNALISTIC NOTES.

Messrs. P. Blakiston's Son & Company have issued a most complete catalogue of medical and surgical works which will be found valuable to any physician. It is pocket size, bound in morocco, with round corners and red edges and contains about 110 pages. It is by far the most practical publication of the kind we have yet seen and may be had of the publishers for the nominal price of 25 cents.

THE *American Journal of Orthopedic Surgery* has changed publishers and will hereafter be printed and published by P. Blakiston's Son & Company, Philadelphia. The subscription price has been reduced from $5.00 to $3.00 a year, and many other advantages will accrue from this change, though its general form and appearance will remain as before.

All matters pertaining to subscriptions or advertisements should be addressed to the publishers. Matters for editorial consideration will be taken up by the editorial committee, which is composed of Dr. R. W. Lovett, 234 Marlboro street, Boston; Dr. H. Augustus Wilson, 1611 Spruce street, Philadelphia, and Dr. A. H. Freiburg, 19 West Seventh street, Cincinnati.

---

THE *Old Dominion Journal of Medicine,* the organ of the medical college of Virginia, and its alumni association, has been turned over to a corporation for publication. It will hereafter be published monthly. Several changes have been made in the editorial staff.

---

THE *Literary Digest,* that admirable weekly compend of world news, has changed its dress, having shed its familiar salmon-pink cover and donned an artistic outside, printed in buff and black on supercalendered paper. This new cover contains two open panels, to be changed every week; one for the contents, and the other to contain a portrait of the man most conspicuously in the public eye during the current week. We are glad to note this indication of prosperity and progress on the part of this staunch weekly magazine.

